# Influencers and COVID-19: reviewing key issues in press coverage across Australia, China, Japan, and South Korea

**DOI:** 10.1177/1329878X20959838

**Published:** 2021-02

**Authors:** Crystal Abidin, Jin Lee, Tommaso Barbetta, Wei Shan Miao

**Affiliations:** Curtin University, Australia; Curtin University, Australia; The University of Tokyo, Japan; Chinese Academy of Social Sciences, China

**Keywords:** coronavirus, COVID-19, influencers, wanghong, press coverage, social media

## Abstract

As COVID-19 broke out across the Asia Pacific from December 2019, media coverage on its impacts proliferated online. Among these discourses, coverage on influencers was prominent, likely as many of the issues arising from COVID-19 contingencies – such as digitalization, public messaging, and misinformation – are cornerstones of this digital economy. In response, this cross-cultural study draws on a corpus of Australian, Chinese, Japanese, and Korean online news articles published between January and May 2020, to understand how local news ecologies were parsing the impacts of COVID-19 on influencers. From the coding of 150 news articles guided by Grounded Theory, this article focuses on the impact of the pandemic on influencers, and influencers’ engagements with and reactions to the pandemic. Our study of individual governments’ past engagements with their influencer industries suggest that local backstories and contexts are crucial to decipher why news angles tend to pitch particular stories on influencers.

## Introduction

As COVID-19 broke out across the Asia Pacific from December 2019, media coverage on the pandemic and its impacts on countries, businesses, society, and individuals proliferated online. Among these discourses, coverage on influencers and the influencer industry was prominent, likely as many of the issues that arose from COVID-19 consequences and contingencies at large – such as digitalization, advertising, precarious income, and public messaging and misinformation – are cornerstones of this digital economy. In this vein, this article aims to review and map out the landscape of issues and tensions that have been captured and popularized by news coverage on influencers and the influencer industry during COVID-19. Specifically, the article draws on a corpus of online news articles published between January and May 2020, focusing on coverage on the markets in Australia, China, Japan, and South Korea; these articles were generally pitched to local audiences, and published in English, Mandarin, Japanese, and Korean, respectively.

Our cross-language and cross-cultural analysis aims to offer an understanding of how different vocabularies for ‘influencer’ and ‘COVID-19’ across the four markets have shaped public understanding of the impact of the pandemic on one segment of the digital economy, and how the politics of press and reporter preferences for select influencer populations, demographics, genres, and platforms – usually guided by clickability and ‘social media logics’ (Van Dijck and Poell, 2013) – have contributed to shaping public perception of the influencer industry at large during the pandemic. The larger contribution of this article aims to query systems of knowledge production and the ‘feedback loop of disproportionate information amplification between journalism and academia’ (Abidin, 2019a: 32), where the usually uninterrogated epistemologies of reporters and press outlets guide and shape media studies of social phenomena, which in turn systemically institutionalize issues spotlighted in the popular media as ‘science’. The specific focus on Australia as the English marketplace and three East Asian markets and languages supports our aim to decenter Anglo-centric discourses, to contribute to decolonizing academic studies on the influencer industry, and to pioneer an ideo-geographic framing of how to document and remember a version of history – in this case, the impact of COVID-19 on the influencer industry in the Asia Pacific.

Like news coverage on ‘millennials’ – who are purportedly ‘killing’, ‘destroying’, and ‘ruining’ dozens of industries and practices, from bar soaps and gyms to loyalty and sex (e.g. Bryan, 2017; Taylor, 2020) – headlines about influencers are similarly low hanging fruit, accessible for public discussion and critique, with the high potential to evolve into trending online topics. As newsrooms and journalism increasingly migrate to digitalization and online-first portals (Benton, 2018; Cottle and Ashton, 1999; Hermida, 2009), reporters are faced with a myriad of pressures: Contents have to be ‘more emotive and shareable’ to appeal to readers (Wilding et al., 2018: 2); outputs have to keep up with the ‘new information asymmetry’ such as the ‘24/7 news cycle’ (Wilding et al., 2018: 5); social networks become key ‘information delivery conduits’ (Heikkilä and Ahva, 2015: 54) and the platforms’ algorithmic logics gatekeep users’ ‘discoverability’ (Lobato, 2018) and access to the information; and competitors participate in creating ‘a favourable news “event”’ to ‘game’ the digital news system and attracted viewership (Mosco, 2017: 139).

Given that influencers are generally ‘digital first personalities’ (Hutchinson, 2019), their followers are also attuned to online trending topics and online news coverage on the industry. Considering these pressures and the ‘social media logics’ – that is, ‘the processes, principles, and practices through which these platforms process information, news, and communication, and more generally, how they channel social traffic’ (Van Dijck and Poell, 2013: 5) – that likeable persona and trending topics facilitate news popularity and visibility, news providers are thus likely to engage in clickbait (Bazaco et al., 2019) and sensationalism (Kilgo et al., 2016) to draw in readership and sustain their ad revenue models. Such digital media practices are exacerbated during the crisis news cycles, where stories that accumulate early ‘social media reactions’ (Stempeck in Owen, 2019) tend to have ‘outsized impact[s]’ and ‘staying power’ in the public imaginary (Owen, 2019). Following from this, this article turns to review and assess the topics and tonalities of news coverage associated with influencers during the COVID-19 news cycles.

In the rest of the article, we will review the role of influencers in the information landscape and specific to the four country markets pre-COVID-19, present the methodology of this study, and present two sections of our findings. The first section focuses on influencer interventions during COVID-19, in relation to messages promoting COVID-19 campaigns and intertwined with misinformation; and the second section focuses on the impacts of COVID-19 on influencers, specifically the state of their income, backlash and criticism, and the need to strategize to cope with the consequences of COVID-19. In closing, we consider how this news coverage has shaped public understandings and public perceptions around knowledge about the influencer industry.

## Role of influencers in the information landscape

In light of COVID-19, some governments have formally enlisted the help of influencers to manage the information landscape. Prominent examples include the Finnish government’s classification of influencers as ‘critical actors’ during the pandemic – alongside ‘doctors, bus drivers and grocery workers’ – as they have been designated to ‘disseminate information on social media’ to make up for the fact that ‘government communication doesn’t reach everyone’ (Heikkilä, 2020). Likewise, the UK government has employed influencers to ‘help spread accurate health information’ especially among younger audiences who ‘may be more susceptible to fake information’ (Pritchard, 2020). Turning to post-COVID-19 recovery, the Indonesian government has similarly set aside around US$5.2 million to engage influencers to ‘promote the country’ and encourage tourism to boost the economy (Gorbiano, 2020). To understand the role of influencers in the four country markets in our study, this section will review the current academic literature on influencer cultures in each country and surmise the governments’ approaches to influencer partnerships pre-COVID-19.

In summary, academic research across the four countries differed in their foci, in reflection of the different pre-COVID-19 partnerships between governments/industry and influencers, and the different mainstream uses of influencers in each country market. In Australia, research had focused on influencers on specific platforms and their role as public relations (PR) actors. This reflected the Australian government and industry’s primary use of influencers as disseminators and endorsers of various sponsored messages to the general public. In China, research had focused on the commercial power of influencers and their contributions to the digital economy. This reflected the Chinese government’s restrictions on citizen discourse online, which confined influencers to gaining popularity and fame through their social selling prowess rather than through other forms of self-expression. In Japan, research had focused specifically on how influencers facilitated reliable information dissemination during times of crisis. Despite Japan’s long history of digital workers and creators (e.g. net idols), the vocabulary and concept of the ‘influencer’ who can transcend genres outside of the traditional entertainment industry was only introduced recently, and as such is still gaining traction in the press and in the academy. In Korea, research had focused on influencers’ progression from blog-based to social media-based platforms, and subsequent issues associated with each stage of this progression, such as misinformation and deceptive advertising. This reflects users’ rapid migration from traditionally popular Korean blog sites like Naver to Silicon Valley social media platforms like Instagram, and emergent issues as a result of changing trends.

### Australia

In Australia, academic research on influencers appears to be platform-specific. Australian blogs have been studied among fashion bloggers as PR actors (e.g. Cassidy and Fitch, 2014), mommy bloggers and monetizing (e.g. Archer, 2019), and feminist and political blogs for advocacy work (e.g. Shaw, 2011, 2012). Australian YouTube cultures have been studied through lesbian, gay, bisexual, transgender (LGBT) influencers who perform care labour and advocacy work (e.g. Abidin, 2019b; Abidin and Cover, 2019), and teens’ career aspirations (e.g. Healy and Cunningham, 2017). Emergent research has also focused on public service media adopting influencer techniques as cultural intermediaries (e.g. Hutchinson, 2017).

Pre-COVID-19, the Australian government had partnered with influencers to promote specific health messages through dedicated social media campaigns – for instance, investing more than AUD700,000 of taxpayers’ money on the Health Department’s 18-month #girlsmakeyoumove campaign (Long, 2018) – but post-campaign assessments revealed inconsistencies between the campaign message and influencers’ track records (e.g. promoting alcohol and diet pills, making rape jokes, expressing homophobia). In 2018, the Australian government banned the use of influencers in its future marketing campaigns (Dawson, 2018) due to the lack of a thorough vetting process (Hickman, 2018). However, influencers were subsequently hired for various government campaigns pertaining to tourism (Parke, 2019) especially after the 2020 Australian bushfires (Jervis-Bardy, 2020).

### China

In China, academic research and public perception of *wǎnghóng* (the Chinese equivalent of ‘influencer’, see ‘methodology’ section below; hereafter *wanghong*) tend to focus on their commercial power (Hu and Zhang, 2017), contributions to the economic market (Cai, 2015; Peng et al., 2019; Sun and Wang, 2019), and their impact on social values and morals especially for young people (Sun, 2012). Research and interest on *wanghong* have also risen alongside the transformation of digital China (Lin and Kloet, 2019), especially due to the development of e-commerce economies facilitated by the likes of Taobao’s live broadcast and the short video platform Douyin. *Wanghong* are generally prized for being ‘consumer opinion leaders’ (Song et al., 2018).

Pre-COVID-19, this almost exclusive focus on *wanghong* and their impact on the economy is in large part due to China’s tight control of the Internet through the China Administration of Cyberspace (CAC) – the country’s highest ranked Internet governance body (Miao and Lei, 2017) – that issues policies to limit citizens’ online expressions and discussions around political issues. As such, *wanghong* in China have emerged as a group primarily comprised of young people who spread impact and leadership in the areas of fashion, entertainment, and consumption, especially on popular digital platforms like Douyin, Kuaishou, Taobao, Xiaohongshu, WeChat, and Weibo (iResearch Consulting, 2018; Wanghong Red Book, 2019).

### Japan

In Japan, academic research has mostly focused on the role of social media and influencers in the aftermath of natural disasters like the 2011 Tōhoku earthquake and tsunami (Hashimoto and Ohama, 2014; Tsubokura et al., 2018). A study on the diffusion of scientific information on Twitter found that the vast majority of tweets concerning the nuclear disaster had been initially produced by a small group of influencers, who have since been deemed important collaborators for diffusing reliable information to the general public (Tsubokura et al., 2018: 2). We can observe an alternative approach to Japanese social media research in the work of anthropologist Gabriella Lukács (2020), whose study explored different categories of female influencers – for example, net idols and bloggers – calling attention to the processes of value extraction that have affected women in the Japanese digital economy.

Pre-COVID-19, the Japanese digital economy had already systematically capitalized on forms of do-it-yourself (DIY) online careers from the 2000s through various platform economies (Steinberg, 2019), even though the vocabulary of the ‘influencer’ was only introduced much later on. Freelance commercial influencers and actors represent a substantial economic resource for digital platforms in Japan. For instance, in 2018, around JPY22 billion was spent on influencer marketing (Digital InFact, 2019). Of this, YouTube was ranked at the top with a market share of 39%, followed by Instagram (27%), and blogging/microblogging platforms (including Twitter; 23%) (Digital InFact, 2019) – a distribution which demonstrates the growing prevalence of visual platforms over the older textual forms.

### Korea

In Korea, studies on the influencer industry appear to be platform-specific. For example, research on blogs has focused on bloggers’ opinion leadership (Kim, 2014), blogger credibility, and customers’ purchase intention (Yun et al., 2012). YouTube has drawn scholarly attention regarding fake news and far-right movements (Chung et al., 2019; Yang, 2020), and studies on Instagram have focused on brand marketing (Kim and Han, 2016). The vocabulary ‘influencer’ has been mostly used and studied in the field of advertising and marketing (Choi and Cheong, 2017; V.I.B et al., 2017), but recently became a buzzword concerning its overt commerciality and related regulatory issues.

Pre-COVID-19, the focus on influencers was on their commercial dealings and transparency (or lack thereof). This coincided with the rapid expansion of the influencer industry, when blogger-driven, text-based influencers on Naver were swiftly outdone and replaced by the more multimedia-driven, visual-oriented influencers on YouTube and Instagram (Cho, 2020; Goodwin et al., 2016; Ryu, 2019). For instance, when the Kim Young-ran Act was instituted in 2017 to ban the bribery of public officials – including journalists – companies turned instead to influencer marketing, using ‘somewhat ordinary people’ (Choi and Cheong, 2017: 48) with some followers on social media (V.I.B et al., 2017) to promote their wares. This has increased concerns and criticisms about deceptive advertising and tax evasion in the influencer industry, as noted in the derogatory expression, ‘인스타 팔이피플’ (Insta *Pal-ee people*, meaning ‘Instagram sellers’) (Jeon and Choi, 2019; Shin, 2018). In response, the Korean government recently announced tightened regulations against influencers’ deceptive advertising and tax evasion (Kim et al., 2020).

## Methodology

We assembled a corpus of online news articles in relation to COVID-19 and influencers, focused on Australia, China, Japan, and South Korea, and published in each market’s native language. Searches were done in incognito mode in mid-May 2020 to exclude the algorithmic impact of our past digital footprints and search histories, and comprised articles dated between January and May 2020. For Australia and Japan, articles were searched and collected on Google, as this search engine is the most used in both countries, at 94.67% uptake (Statcounter, 2020a) and 77.4% uptake (Statcounter, 2020b) respectively, as of May 2020. These Google searches were conducted on the default general search tab as opposed to the dedicated ‘News’ tab to limit Google search’s structural bias towards ‘prominent’ news publishers that were particularly visible in the latter (Rankovic, 2019). For China, articles were searched and collected on Baidu, which is the dominant platform in the search engineering market in China, after Google left Mainland China in 2010 due to issues of internet censorship (Waddell, 2016). Baidu holds a 76.05% market share domestically (CIW Team, 2017), and ranks first in China’s search engineering field, and second on a global scale (Alexa, 2020). For Korea, the Korean search engine Naver was selected for being the most used search engine in the country (Internet Trend, 2020). Articles were searched and collected on Naver’s dedicated online news service, Naver News, which is the most popular news distribution channel online in Korea (Choi and Lee, 2017). Naver search algorithms categorize every search and show the results under several tabs, such as ‘News’, ‘Internet cafe’, and ‘Shopping’. We primarily gathered news articles that were automatically streamed under the ‘News’ tab (Park, 2010), but cross-checked with search results on the other tabs to surface any missing items.

### Search terms

Our first keyword pertained to ‘influencers’. For Australia, Japan, and Korea, we searched for the keyword ‘influencer’ and for China we searched for ‘网红’ (wǎnghóng). In Australia, ‘influencer’ is the most used and levelling catch-all phrase to refer to internet celebrities, digital creators, and platform-specific microcelebrities such as YouTubers, Instagrammers, and the like. Likewise for Japan, ‘インフルエンサー’ (influensā) is the most accurate and direct transliteration of the English ‘influencer’, which focuses on prominent social media users and is platform-agnostic. For Korea, ‘인플루언서’ (influencer) is also part of the everyday parlance to refer to such social media users. Although there are equally popular alternatives such as ‘콘텐츠 크리에이터’ (contents creators) and ‘파워 블로거’ (power blogger), both were excluded to maintain the consistency of our data sets in this cross-cultural study. For China, ‘网红’ (wǎnghóng) translates to ‘internet red’ and is the most popular catch-all expression for ‘internet celebrity’, ‘microcelebrity’, and ‘online influencer’.

Our second keyword pertained to ‘COVID-19’. For Australia, we searched for ‘COVID-19’, ‘coronavirus’, or ‘pandemic’ and ‘Australia’ as the three variants were the most used words to refer to the 2019-nCoV novel coronavirus and the evolving situation in the country. For China, we searched for ‘疫情’ (yìqíng) and ‘冠状病毒’ (guānzhuàng bìngdú); the former refers to both an ‘epidemic’ and a ‘pandemic’, and the later is an abbreviation of two words – ‘新型冠状病毒’ (xīnxíng guānzhuàng bìngdú) and ‘新冠状病毒’ (xīn guānzhuàng bìngdú) – that refer to the ‘coronavirus’. The search term ‘COVID-19’ was excluded as it tended to be used only in professional medical papers and reports in the Chinese context. For Japan, we searched for ‘コロナ’ (korona), ‘新型コロナ’ (shin-gata korona), and ‘コロナ禍’ (korona-ka), which translate to the generic term ‘corona’, the virus-related term ‘new coronavirus’, and a term reflecting the social implications of the epidemic ‘corona crisis’. For Korea, we searched for ‘코로나’ (corona) and ‘팬데믹’ (pandemic), which translate to ‘corona’ and ‘pandemic’, as these were the most used vocabulary to refer to COVID-19; Korean media generally refers to the virus as ‘corona virus’ and the English word ‘pandemic’ has been adopted into the everyday vocabulary of (post-)COVID-19 Korea.

### Search parameters

On all the search engines used, each page presented an average of 10 results. Our corpus of articles was compiled from the first 10 pages of each search engine. We excluded a selection of articles based on the digital media norms of each country, pertaining to press releases, duplicates, multimedia, paywalls, blogposts, and authorship. In general, press releases that were paid promotions, duplicates and reposts of news articles across a longtail of smaller outlets, and audio-only and video-only news articles without any text were excluded. Considerations for paywalls, blogposts, and authorship varied by country: (1) For Australia and Korea, most reputable news sites are open access and preferred by readers (Cheik-Hussein, 2019; Choi and Lee, 2017), thus paywalled news sites with gated access were excluded. In Japan, the norm is for viewers to register or subscribe to news media portals – although most of these are not paywalled – thus gated subscription articles were included but paywalled ones that could not be accessed freely were excluded. In China, the most popular and common way people consume information and news is through free online websites and public accounts of WeChat, so paywalled and subscription websites are excluded in this study; (2) For Australia, the blog platform *Medium* is often used by reputable companies and outlets to publish news articles, so these were included. Similarly for China, it is the norm for verified companies and news websites to publish news on WeChat public accounts that resemble blogposts, so these were included. For Japan and Korea, blogposts were not usually deemed valid news sources and thus excluded; (3) The Australia, Japan, and Korea samples excluded news articles whose authors could not be verified, unless they were intentionally represented as a ‘team’ or ‘staff’ of the platform. However, for China pen names and the use of pseudonyms is the norm even for news articles, so these were included in the study.

In total, we surveyed 150 articles: 42 in English for Australia, 39 in Chinese for China, 31 in Japanese for Japan, and 38 in Korean for Korea. The next section maps out the open, axial, and close codes that were derived from the headlines and news articles, guided by a grounded theory approach (Glaser and Strauss, 1967).

## Overall trends in news coverage on influencers during COVID-19

After analyzing the headlines and story of the news articles, seven open codes emerged across our corpus: Income loss, Backlash, COVID-19 campaign, Misinformation, Influencer strategy, Brand leverage, and Industry shifts. For ‘Income loss’, close codes focused on ‘Clients/Brands’ and ‘Followers/Fans’; for ‘Backlash’, close codes focused on ‘Safety guidelines’, ‘Safety equipment’, and ‘Goods/Services’; for ‘COVID-19 campaign’, close codes focused on ‘Isolation regulations’, ‘Hygiene habits’, ‘Social behaviours’, ‘Formal campaigns’, and ‘Trends’; for ‘Misinformation’, close codes focused on ‘Causation’, ‘Reduction’, and ‘Topics’; for ‘Influencer strategy’, close codes focused on ‘For self’, ‘For brands’, ‘For followers’; for ‘Brand leverage’, close codes focused on ‘Targeting investors’, ‘Targeting influencers’, ‘Targeting new digital media formats’, ‘Targeting consumers/customers/viewers’, and ‘Types of brands/clients’; and for ‘Industry shifts’, close codes focused on ‘Brand preferences’, ‘Content production’, ‘Content format’, ‘Follower preferences’, and ‘Type of influencers’. Full-coding categories from our analysis are in the Appendix (Table 1). In this article, we will focus on five open codes touching on the impact of the pandemic on influencers (i.e. Income loss, Backlash), and influencers’ engagements with and reactions to the pandemic (i.e. COVID-19 campaign, Misinformation, Influencer strategy).

Across the 150 articles, some trends emerged with regards to the demographic of influencers represented. There were slightly more women than men in the reporting, with 104 female and 88 male influencers mentioned. The age of influencers was rarely mentioned in the press coverage with the exception of articles from Japan, but those presented were primarily in their 20s to early 30s. In total, 25 platforms were mentioned in our corpus, including Silicon Valley-based apps and local apps, such as WeChat (China), Naver (Korea), and Afreeca TV (Korea). In total, 98 out of 140 mentions of platforms focused on those that were visually oriented photo or video sharing sites; Instagram was the most frequently mentioned (n = 49), followed by YouTube (n = 32), and TikTok (n = 12) (Table 2 in Appendix). The news coverage tended to focus on influencer content genres that were feminized or dominated by women influencers, such as entertainment (n = 32), followed by fashion and beauty (n = 25), lifestyle (n = 24), food (n = 11), and health (n = 11). Other genres that were reported on include entrepreneurship (n = 6) and family (n = 3) (Table 3 in Appendix).

## Impact of the pandemic on influencers

### Income

Some of the news coverage on Australian influencers highlighted their income loss due to the pandemic. Influencers cited feeling that ‘years of hard work’ had ‘gone down the drain’ (Galea, 2020) due to cancelled business engagements. Restrictions from social distancing measures also impacted influencers’ ability to produce content, given that much of their job requires them to be mobile to travel or participate in public events. For other influencers, travel content that had already been prepared and queued for publication was pulled by clients, such as tourism-related businesses that did not want to advertise their services during the pandemic when potential customers were unable to travel. Australian influencers were also reported to ‘slash their rates’ and ‘dro[p] their fee’ for social media posts by up to 50% (Wilkie, 2020), to accommodate clients’ reduced budgets and ensure that they could maintain at least some income stream. In a prominent example, Australian x-rated influencer Billie Beever reportedly uploaded a TikTok video of herself crying at the prospect of being unable to afford rent due to a huge drop in income on the subscriber platform OnlyFans. As social distancing measures did not allow her to pivot to sex work during the pandemic, she expressed that she had ‘nothing else going’ for income (news.com.au, 2020). The Chinese corpus similarly reported some income loss due to declining advertising demand among *wanghong*, with a market research company citing that one-third of *wanghong* have been impacted (Nan and Chen, 2020).

However, other Chinese reports posit that *wanghong* have become more influential as viewer traffic and engagement have increased in light of stay-home advisories. In particular, online live streaming was experiencing a boom and being a *wanghong* had been deemed one of the most attractive jobs during the pandemic (Shao and Qin, 2020). The coverage in Japan did not focus on income loss. Instead, an article asserted that lucrative collaborations between influencers and brands were growing during the pandemic (Ito, 2020). Our results recursively portrayed influencer marketing as the key for brands to adapt to the new patterns of ‘stay-at-home consumption’ that characterized the pandemic (Kabutan News, 2020). A candid statement in the aforementioned article reads: ‘Income loss due to corona . . . they [top influencers] might not be bothered at all’ (Ito, 2020, translated by authors), suggesting that unlike most people whose livelihoods were impacted by COVID-19 on some level, some influencers appear to be shielded. In a similar vein, some of the coverage on Australian influencers suggested that the impact on income was uneven across the genres, as those who primarily promote beauty and fashion are still able to produce content from home to advertise e-commerce clients. Our Korean corpus did not mention income at all, possibly due to the sensitivities around transparency of income and taxation mentioned earlier.

In this section, the Australian corpus tended to highlight the economic impact of the pandemic on influencers, emphasizing their income loss, strategies for recuperating losses and soliciting partnerships with clients, and a general sense of helplessness due to restrictions on mobility, and therefore, content creation. This scenario reveals that Australian influencers have overtly relied on client engagements for their income and have yet to diverse their income streams – in other words, Australian influencers appear to have ‘put all their eggs in one basket’, which was swiftly toppled over by the contingencies of COVID-19. On the contrary, Chinese influencers who were already concentrating on online e-commerce and social selling experienced a boom during COVID-19, as followers and customers increased their online viewing and purchasing activities. This was also the case for Japanese influencers to a certain extent, who were once again framed as key information mediators during a crisis, this time for brands and the industry alongside their previous public service roles during the floods. We note that the absence of press coverage on influencers’ income loss in Korea is likely due to the recent controversies over deceptive advertising and tax fraud.

### Backlash

Many Australian, Chinese, and Korean influencers received criticism for jeopardizing COVID-19-related advisories and regulations; the corpus of Japanese articles did not report on the backlash faced by influencers. For instance, influencers in Australia and *wanghong* in China received backlash for continuing to post about their travels, and in some instances, even continuing to travel during the period of mass self-isolation and safe-distancing measures. Followers were also critical towards some Chinese *wanghong*’*s* personal values as posts depicting influencers pretending to eat bats – in reference to early speculation of the origins of COVID-19 – were a distasteful way to attract attention and bait viewership. In Korea, a fashion influencer Imvely was mentioned in two news articles that reported on public personalities (including K-pop stars and actresses) who threw a big birthday party at a café in Seoul in May, around the time a new COVID-19 cluster had emerged around clubs, bars, and cafes in downtown Seoul. The personalities were called out for being irresponsible and ignoring social distancing advice, in their pursuit of fun and pleasure. However, it should be noted that in the Korean influencer industry, Imvely already had a track record of notoriety from past intransigence – for instance, selling and advertising mouldy food on her Instagram (Kwak, 2019) – and was thus an easy target for tabloidesque news publications. For the most part, Korean influencers did not cop too much backlash for their behaviours during COVID-19.

Apart from criticism on their obvious offences, the backlash against the influencers during the pandemic also extends to their tone-deafness. Some Chinese *wanghong* were called out for posting entertainment content featuring lighthearted dancing and singing, which viewers felt was inappropriate in light of mass suffering in the country. Likewise, Korean influencer Imvely was criticized for her ‘insincere attitude’ and for continuously advertising her products on Instagram without any formal apology to the public for her ‘irresponsible behavior’ (Kim, 2020d). A British-Australian actor influencer was also outed on social media by the company from whom he had asked for freebies in lieu of promotional content on social media. In Australia, two articles focused on health and fitness influencer Jessica Pinili who, when placed in a five-star hotel to serve out her 2-week mandatory quarantine period after returning from Bali, posted an Instagram story criticizing the Australian government for her poor living conditions. She complained about not having ‘access to a balcony’, lacking ‘fresh air’, and feeling ‘worse than being a prisoner’, and called this a ‘human rights’ issue despite her quarantine stay being sponsored by the government (Sanders, 2020). To this, Australians criticized the influencer for being a ‘princess’ and ‘spoiled brat’ and flooded her social media with negative feedback (Brook, 2020).

Some influencers were also criticized for making light of the pandemic. Some Australian influencers and Chinese *wanghong* were chided for posting fashionable photos of mask-wearing, or for reappropriating masks as a fashion statement. This was deemed inappropriate and insensitive, especially in light of the mass shortages that occurred globally in the early stages of the COVID-19 outbreak. One commenter on an Australian influencer’s post noted their displeasure: ‘I’m so sorry I really like you guys, but doctors and nurse are in desperate need for those masks. So even if you need them it should not be turned out as an aesthetic picture for your feed’ [sic] (Booth, 2020). However, a handful of commenters perceived such influencers’ actions conversely, and praised them for promoting and normalizing mask-wearing in public as a safety measure.

In this section, influencers across all country markets received backlash and were criticized for a variety of faux pas and infringements. This reflects the heightened policing of conspicuous online displays by influencers and ordinary citizens, in line with users and followers spending much more time online consuming influencer content, and the rising sensitivities of followers as the pandemic extended. As we note earlier in this article, this increased coverage on influencer scandals and bad publicity also has the tendency to be tabloidesque moves by traditional and digital media outlets to attract viewership, and may at times serve as mere clickbait, thus exaggerating incidents and blowing issues out of proportion.

## Influencers’ engagements with and reactions to the pandemic

### COVID-19 campaign

Some influencers responded to COVID-19 by initiating vernacular trends to promote good social behaviours, such as social distancing and good hygiene practices. In China, local reports took interest in a group of foreign grandmothers based abroad, with two articles focused on how they used Instagram and YouTube to urge people to commit to hygiene practices, and to stop discrimination or racism (CCTV News 2020; Han, 2020). Such influencers had emerged organically during the pandemic as their follower base grew alongside their growing virality, and the Chinese elderly *wanghong* were celebrated because ‘the optimism delivered by them [was] more valuable and important than ever’ (Han, 2020, translated by authors). Australian and Korean influencers were similarly noted for promoting social distancing, handwashing techniques, mask use, and responsible cough etiquette. In Australia, this included handwashing dances and memes on the short video app TikTok, and viral retweet chains on Twitter that were estimated by the government to be worth around ‘$30 million’ in advertising (Rose and Alison, 2020). This string of positive PR angles on Australian influencers in partnership with the government were likely to make up for the bad aftertaste of the past failures mentioned earlier.

Several articles pointed to influencers participating in formal campaigns with health agencies and governments to assist in COVID-19 recovery efforts. In Korea, many influencer COVID-19 campaigns were initiated or sponsored by the Korean government or international organizations, especially targeting young people. For instance, the Korean government partnered with animation companies and child influencers such as Awesome Haeun (YouTuber) to introduce social distancing rules with songs and dancing (Jung, 2020). In a similar manner, the Asia-based media agency Millenasia and UNESCO Global Education Coalition collaborated on a YouTube music video featuring K-pop singers to promote COVID-19 prevention rules and to encourage students and children to ‘stay strong’, while practising social isolation during this time (Ha, 2020). In Japan, multiple articles covered the interventions of influencers individually promoting self-isolation and handwashing practices. In particular, a collaboration video between the governor of Tokyo, Yuriko Koike, and one of the most influential YouTubers in Japan, Hikakin, discussed the risks of COVID-19 and was reported in the news (Kōkōsei Shinbun Henshūbu, 2020). An article praised the partnership as tangible proof of the influence of YouTubers in contemporary society: ‘[YouTubers have become] something close to public figures, and as such, are playing a role in casting information to prevent the epidemic from spreading’ (Kamada, 2020, translated by authors). In Australia, influencer marketing platforms have also collaborated with influencers to promote healthy messaging around well-being and health, in partnership with the World Health Organization (WHO) and National Mental Health Commission (e.g. the ‘#InThisTogether’ campaign (Life in Mind, 2020)). Around 80,000 influencers in Australian company Tribe’s network were invited to ‘submit free content that includes advice and “hacks” on social distancing’ (Cheik-Hussein, 2020) that were subsequently boosted on social media to reach a million people under 24 hours.

Some influencers also called out bad social behaviours, such as anti-Asian racism and xenophobia, and panic buying and hoarding. In particular, Asian-Australian influencers were reported to experience racist and xenophobic attacks, ranging from hate comments on their social media – for example, ‘stop eating bats’ (Wilson, 2020) – to uncomfortable interactions in public. In response, some of them have taken to acting as ‘ambassadors of both cultures’ (Abidin in Robinson, 2020) by bringing awareness to such behaviour and producing commentary and content to educate their followers.

In this section, our findings reveal that influencers in all country markets were sought after to promote COVID-19 etiquette and normalize new behavioural norms. This suggests the efficacy of influencers in information dissemination and control in the online space, as citizens worldwide relied on the Internet for information and entertainment during prolonged self-isolation and social distancing measures.

### Misinformation

In our corpus, the topics that contended with misinformation include health advice (what to do and what not to), vaccinations (whether they are preventive or cause COVID-19), 5G (apparently causing COVID-19), and Bill Gates (apparently planting COVID-19). For the most part, articles that discussed influencers and misinformation focused on their role in the infodemic.

Some influencers were reportedly responsible for spreading misinformation. In Australia, an article called out a former reality TV star-turned-Instagram influencer Amanda Micallef for encouraging followers to ‘ignore lockdown restrictions’ and sharing unverified information that COVID-19 was ‘a ruse to get rid of physical money’ (Gordon, 2020). Another Australian article pointed to the conspiracy theories flamed by ‘anti-vaccination influencers’ (Vinall, 2020). In response, the President of the Royal Australian College of General Practitioners, Dr Harry Nespolon, cautioned influencers to remain ‘silent on the topic’, to acknowledge that they are ‘not epidemiology experts’, and to allow medical experts to lead the advisories and commentating (Mara, 2020). In China, one article pointed out misinformation spread by *wanghong* who had apologized to Europe for the pandemic, on behalf of China, through a Weibo post – this was deemed to be misinformation by the Chinese state as there was still not sufficient evidence that the virus had indeed originated in China (Minzihui, 2020). The absence of misinformation from Chinese *wanghong* may be attributed to governance by the CAC mentioned earlier in this article, and the government’s strong-handed control and censorship that limits public political engagement even in disaster communications. Furthermore, it is suggested that there is a weak civil society in China (Xu, 2017), wherein netizens lack the literacy and capacity to engage in advocacy campaigns, unless they are organized and promoted by the government.

In Japan, two articles referred to influencers as the causes of misinformation (Abema Times, 2020; Manabe, 2020). While no specific influencer was named, one article stated that ‘the incorrect belief that alcohol disinfection does not work with SARS-CoV-2 has spread mainly because of influencers’ (Manabe, 2020). Another article drew a parallel between the spread of misinformation by influencers after the 2011 Tōhoku earthquake and during the COVID-19 pandemic (Abema Times, 2020). Journalists cautioned that on social media, ‘problems arise when influencers are the sources of information’. In this case, ‘false rumors end up attracting many people, receiving tens of thousands of retweets and likes’ (Abema Times, 2020, translated by authors). In Korea, contrary to increasing concerns over some YouTubers spreading harmful misinformation about COVID-19 (e.g. Kim, 2020b), only one article covered influencers’ spread of misinformation, but this was discussed more broadly in the global context, and no Korean incidents were named (Kim, 2020c). This is likely because of the neutral stance of the term ‘influencer’ in Korea, as opposed to other platform-specific terms with derogatory nuances (e.g. ‘YouTuber’ with a reputation for distributing misinformation).

In our analysis, there were several articles that hinted at the potential for influencers to reduce and curb misinformation, by soliciting their cooperation to promote information verified by the government and health agencies. This has been reviewed in the preceding section in which influencers were actively participating in various COVID-19 campaigns. Only one article specifically named the efforts of influencers to combat misinformation, in a Japanese example of influencers collaborating with LINE, the most popular instant messaging platform in the country, to combat misinformation following the WHO guidelines (Line corp, 2020).

In the previous section, we note the efficacy of influencers in aiding with good and healthy information control. However, the reverse was also true for the Australian, Chinese, and Japanese markets, where influencers were also found to be peddling in misinformation, whether intentional or not. The apparent absence of reporting on influencers and misinformation in Korea is likely due to the implications and innuendos of different vocabularies in the highly localized context. As we note earlier in the article, specific terms referring to Instagram influencer sellers (‘인스타 팔이피플’ or Insta Pal-ee people), were more likely to be related to negative press.

### Influencer strategies

In general, reports from Australia, China, and Japan present four categories of strategies for influencers themselves (two types), for the brands and clients with whom they partner, and for their followers; the Korean corpus did not touch on influencer strategies. First, many influencers shared their personal experiences in daily diaries or mental health tips as their own coping strategy, and to foster a sense of solidarity among others in self-isolation. Japanese influencers primarily shared their personal experiences during the pandemic through interviews in news articles or by writing such articles themselves. In an interview, an influencer openly addressed the issue of mental health and distress caused by social isolation, specifically from the point of view of sexual minorities (Shinoda, 2020).

Second, to cope with income loss and the constraints around content production, some influencers extended their personal brands and commercial endeavours by branching out into e-commerce, participating in merchandising online, and starting other related businesses. An Australian brand management agency, Slyletica, revealed that they had a 200% increase in ‘entrepreneurs and influencers wanting to start their own activewear labels’ (Zhou, 2020) during the pandemic. Other influencers were reportedly considering subscription models and membership programmes for followers that would ‘offer a dependable source of revenue separate from the volatile ad market’ (Perelli and Whateley, 2020), and still some influencers are looking into ‘consulting, teaching, and coaching’ as ‘alternative revenue streams’ (Perelli and Whateley, 2020).

Third, some influencers committed to doing pro-bono work for brands and clients, or free work and charity for various causes. For instance, Chinese *wanghong* were documented providing expertise and assisting farmers in rural areas to sell local products. Given that China is still largely an agricultural country, many farmers encountered economic difficulties during the pandemic because products could not be distributed as easily. As such, some *wanghong* helped local farmers to sell their products online by advertising these products in their livestreams. Alongside *wanghong*, some cadre and local government employees also started to initiate such online collaborations to assist rural economies, and have been lauded as a new or special type of *wanghong* who differ from the stereotypical young, fashionable, and eye-catching personalities. As a result, *wanghong* has evolved to be a buzzword or catchphrase for anyone in China who can attract attention and become famous online.

Finally, to sustain follower interest and engagement, several influencers turned to creative strategies in their content generation. In light of restricted mobilities, Chinese *wanghong* who may usually record their content outdoors with the help of professional equipment and assistants have since pivoted to solo indoor shoots. Across the genres, their contents have also shifted to focus on their everyday lives, or the documentation of latest updates or less-reported information about pandemic. For instance, local influencers in Wuhan were reported to be making vlogs about the city scene and people’s lives during pandemic, and some foreign *wanghong* in China had begun to produce content collating updates on how other countries have been confronting the pandemic. For instance, Fan Wu, an Overseas Chinese student, became a *wanghong* for charting the growth of coronavirus cases on Twitter, in a bid to help UK followers better understand the epidemic. An Australian fashion influencer who lives in Paris had also pivoted to creating a weekly video series to catalogue their life during lockdown and ‘tak[e] part in a worldwide collective social experience’ (Liu J, 2020). In a similar vein, multiple Japanese articles featured influencers who appear to be making use of their social capital (e.g. acquaintances) and cultural capital (e.g. language knowledge) to connect with people living in other countries and report on their experiences of the pandemic. Japanese influencers were also celebrated for making extra effort to communicate with their followers during the pandemic. A recurrent example is that of Naomi Watanabe, the most followed Japanese influencer on Instagram, who was praised for her ‘outstanding social media skills during the corona crisis’ (A4studio, 2020), through the intimate sharing of her everyday life that entertained and encouraged fans. However, such extensive coverage in the Japanese media might be mirroring a bias towards focusing on influencers with a large mass of followers to attract viewership.

In this section, influencers in Australia, China, and Japan were unanimously strategizing to recover from their economic slump through a repertoire of creative methods, reflecting the extensive impact of COVID-19 on various businesses and industries worldwide. While there was no reportage on this for the Korean market, this was once again likely due to reporters and news outlets using other localized vocabulary to refer to platform- or genre-specific influencers in the country.

## Conclusion

In this article, we have explored the different issues and tensions in which influencers seem to be embroiled across the four countries during COVID-19. While the English vocabulary ‘influencer’ appears to be globally adopted and used across the world, how the concept of ‘influencer’ is perceived and understood, especially concerning the global pandemic situation, was varied in relation to local contexts. This signifies a need for cross-cultural comparative studies that decolonize and dewesternize academic discourses that have evolved around English-speaking and/or Global North countries. Given that the contemporary mediascape changes and responds to global and local contexts, it is crucial to note the plurality of *cultures* when we conceptualize cultural phenomena.

From our analysis, we observed the attention given to influencers across the four countries, concerning especially their socio-cultural impact. Across the country markets, the utility and impact of influencers have become especially crucial during a pandemic like COVID-19, when uncertainty is extreme (and people look to key opinion leaders for information and guidance), precarity is on the rise (and people look to entertainment for placation), and physical social interactions are restricted (and people turn to digital spaces as their first port of call for all activities). For this reason, governments and health organizations have sought partnership with influencers to assist in COVID-19 recovery efforts and to pursue global solidarity. Compared to pre-COVID-19 times, influencers were mobilized at a larger scale for a wider variety of uses across topics and genres. Influencers themselves had also endeavoured to engage with the society, by doing pro-bono work, promoting social distancing and hygienic practices, and calling out problematic phenomena such as racism. However, we note that the potential of influencers to serve as effective amplification platforms is contentious and complicated when these individuals intentionally or unwittingly peddle in generating and spreading misinformation.

However, influencers appear to be convenient topics to feed cycles of tabloidesque news which rely on clickbait and sensationalism to appeal to viewers. In line with the luxurious, flamboyant, and aesthetic stereotypes of influencers (Abidin, 2014), whether or not they cope with the pandemic situation becomes newsworthy, and can easily draw attention from viewers from whom news outlets depend on site traffic and ad revenue (Myllylahti, 2019). Influencers’ episodes pertaining to COVID-19 (e.g. complaining about safety measures, ignoring social distancing advice) make for easy celebrity gossip, especially when they are generalized as and reduced to a privileged class of young people who are unable to empathize with the severity of actual struggles outside their picturesque ‘Instagram bubbles’. In a similar vein, how COVID-19 affects influencers’ economies is newsworthy in the time where the global economy is shrinking and many industries are undergoing economic turmoil, as evident in the Australian and Japanese cases.

The attention economy that underscores this recent news coverage on influencers during COVID-19 also shapes viewer perceptions of the role and value of an ‘influencer’; as evidenced, most of the news coverage has been organized around visual platforms like Instagram, and feminized genres like fashion and travel. While the actual practices and concepts of an ‘influencer’ is broad – comprising people in various genres on various platforms and exhorting various social values – popular notions of influencer cultures are still largely shaped by news coverage that tends towards sensationalism (Kilgo et al., 2016), gendered self-visualization (Duffy, 2015), and pleasure-centred self-display (e.g. drinking photos) (Goodwin et al., 2016). As such, it is hoped that this cross-cultural and cross-lingual study has laid bare some of the local backstories and crucial contexts needed to corroborate and decipher the popular understandings of influencer cultures, largely manipulated by the selective foci of reporters and outlets under a regime of traffic-dependent web media. In future work, the authors will focus on the epistemologies behind transliteral understandings of influencer vocabularies and concepts situated in different cultural contexts in the Asia Pacific region, and invite other scholars to similarly pursue a dewesternizing and decolonializing of influencer studies.

## Appendix

**Table 1.** Full list of codes from our analysis.

Open codes	Axial codes
1. Income loss	Client/brand
	Followers/fans
2. Backlash	Safety guidelines
	Safety equipment
	Goods/services
3. COVID-19 campaign	Isolation regulations
	Hygiene habits
	Social behaviours
	Formal campaigns
	Trends
4. Misinformation	Causation
	Reduction
	Topics
5. Influencer strategy	For self
	For brands
	For followers

**Table 2.** Types of platforms mentioned and reported on in our corpus.

	Silicon Valley apps (e.g. Facebook)	Local apps (e.g. WeChat)
Image-focused (e.g. Instagram, Pinduoduo)	49	4
Video-focused (e.g. YouTube, Douyin, TikTok, Netflix)	37	30
Text-focused (e.g. Twitter, blog)	12	8

**Table 3.** Full list of genres mentioned in our corpus.

Genre	Number of mentions
Entertainment	32
Fashion-beauty	25
Lifestyle	24
Food	11
Health	11
Entrepreneurship	6
misc. (e.g. sex, wildlife, pet)	4
Family	3
